# Supplementary material for: Discovery proteomics in aging human skeletal muscle finds change in spliceosome, immunity, proteostasis and mitochondria
Source: eLife. 2019 Oct 23;8:e49874. doi: 10.7554/eLife.49874 (PMC6810669; doi:10.7554/eLife.49874)
Supplement: Figure 1—source data 1. — Participants are classified into five different age groups. Gender: M is Male, F is Female; the number of participants is indicated. Age is indicated in years as mean and standard deviation (SD ±) for each age group. Race: number of participants is shown on the left and race is shown in italics; C is Caucasian, AA is African American, and A is Asian. Body Mass Index (BMI) is expressed as mean and SD (±) for each group. p-Value is calculated by one-way ANOVA with Kruskal-Wallis test. Race is analyzed by Chi-square test. *p-Value calculated from linear regression model, gender adjusted. ± Knee Extension Isokinetic Strength (KEIS) (300/sec; Nm). †Physical activity is calculated from self-report involvement in weight circuit, vigorous exercise, brisk walking and casual walking and summed as high-intensity physical activity hours per week. This is further categorized into 0 (not active), 1 (moderately active), 2 (active), and 3 (highly active) and expressed as mean of categorical variables (0,1,2,3) ± SD. [file elife-49874-fig1-data1.docx]

**Figure 1-table supplement 1**

| **Age Group** | **20-34** | **35-49** | **50-64** | **65-79** | **80+** | ***P*-value** | **R2** |
| --- | --- | --- | --- | --- | --- | --- | --- |
|  | (*n*=13) | (*n*=11) | (*n*=12) | (*n*=12) | (*n*=10) | -- | -- |
| **Age (yr)** | 27.2 ± 3.3 | 41.3 ± 4.5 | 57.1 ± 4.7 | 70.3 ± 2.3 | 82.4 ± 2.4 | -- | -- |
| **Gender** | *M*8, *F*5 | *M*7, *F*4 | *M*7, *F*5 | *M*8, *F*4 | *M6*, *F4* | -- | -- |
| **Education (yr)** | 16 ± 3 | 14 ±3 | 14 ± 2 | 16 ± 2 | 17 ± 2 | 0.3305 | -- |
| **Race** | 9*C*, 2*AA*, 2*A* | 5*C*, 6*AA* | 8*C*, 4*AA* | 10*C*,1*AA*,1*A* | 9*C*,1*AA* | 0.0958 | -- |
| ***BMI, kg/m2** | 25.9 ± 2.8 | 26.4 ± 2.6 | 26.6 ± 3.2 | 26.4 ± 2.4 | 25.2 ± 3.9 | 0.3458 | 0.007 |
| **Height (cm)** | 172 ± 11 | 177 ± 10 | 169 ± 4 | 172 ± 11 | 172 ± 6 | 0.3985 | -- |
| ***Weight (kg)** | 76 ± 10 | 81 ± 9 | 77 ± 12 | 75 ± 13 | 73 ± 16 | **1.74E-05** | 0.34 |
| ***Waist Circumference (cm)** | 82 ± 7 | 87 ± 7 | 90 ± 11 | 92 ± 11 | 92 ± 13 | **6.32E-06** | 0.39 |
| ***KEIS (left) ±** | 192 ± 31 | 208 ±55 | 200 ± 71 | 165 ± 62 | 130 ± 42 | **4.29E-07** | 0.40 |
| ***KEIS (right) ±** | 194 ± 38 | 220 ± 65 | 194 ±78 | 169 ± 53 | 147 ± 57 | **2.41E-07** | 0.41 |
| †**Physical Activity** | 1.8 ± 1.4 | 1.8 ± 1.3 | 2 ± 1.1 | 2.3 ± 1 | 1.5 ± 1.1 | 0.5145 | -- |

**Figure 1-table supplement 1.** **Baseline Characteristics of the GESTALT Skeletal Muscle Participants.** Participants are classified into 5 different age groups. Gender: *M* is Male, *F* is Female; the number of participants is indicated. Age is indicated in years as mean and standard deviation (SD ±) for each age group. Race: number of participants is shown on the left and race is shown in italics; *C* is Caucasian, *AA* is African American, and *A* is Asian. Body Mass Index (BMI) is expressed as mean and SD (±) for each group. *P*-value is calculated by 1-way ANOVA with Kruskal-Wallis test. Race is analyzed by Chi-square test.

******P*-value calculated from linear regression model, gender adjusted.

**±** Knee Extension Isokinetic Strength (KEIS) (300/sec; Nm).

**†**Physical activity is calculated from self-report involvement in weight circuit, vigorous exercise, brisk walking and casual walking and summed as high-intensity physical activity hours per week. This is further categorized into 0 (not active),1 (moderately active), 2 (active), and 3 (highly active) and expressed as mean of categorical variables (0,1,2,3) ± SD.
